# Indirect somatic embryogenesis of purple coneflower (*Echinacea purpurea* (L.) Moench): a medicinal-ornamental plant: evaluation of antioxidant enzymes activity and histological study

**DOI:** 10.1007/s11033-020-05713-y

**Published:** 2020-08-17

**Authors:** Maryam Dehestani-Ardakani, Mohadeseh Hejazi, Kazem Kamali Aliabad

**Affiliations:** 1 Department of Horticultural Science, Faculty of Agriculture & Natural Resources, Ardakan University, P.O. Box 184, Ardakan, Islamic Republic of Iran; 2 Medicinal and Industrial Plant Research Institute, Ardakan, Islamic Republic of Iran; 3 Department of Soil Science, Natural Resources Faculty, Yazd University, Yazd, Islamic Republic of Iran

**Keywords:** Benzyl adenine, Coconut milk, Casein hydrolysate, Callus, Naphthalene acetic acid

## Abstract

Purple coneflower (*Echinacea purpurea* (L.) Moench) is a widely used medicinal and ornamental plant. In the present study, the callus embryogenesis was examined using benzyl adenine (BA) at three levels (3, 4, 5 mg L^−1^), 1-Naphthalene acetic acid (NAA) at three levels (0.1, 0.2 and 0.5 mg L^−1^) with or without activated charcoal (1 g L^−1^), coconut milk (50 ml L^−1^) and casein hydrolysate (50 mg L^−1^) in the MS (Murashige and Skoog 1962) medium. The embryogenesis indirectly occurred with the production of callus. The calli were observed in three forms: undifferentiated, embryogenic and organogenic. The embryogenic calli were dark green and coherent with a faster growth rate. The highest embryogenesis (100%) and embryonic regeneration (plantlet production) were obtained in the combined BA + NAA treatments with the activated charcoal, coconut milk and casein hydrolysate. However, the combined treatments of growth regulators failed to produce somatic embryos without the use of coconut milk and casein hydrolysate. The maximum amount of protein, peroxidase and catalase activity of embryogenic calli (2.02, 1.79 and 6.62ΔOD/Min/mg.protein, respectively), and highest percentage of acclimatization success (29.3% of plants) were obtained in the combined treatment of 5 mg L^−1^ BA + 0.5 mg L^−1^ NAA + activated charcoal + coconut milk + casein hydrolysate. The highest amount of chlorophyll content (33.3 SPAD value) and growth characteristics of acclimatized plantlets were observed in the media containing 3 mg L^−1^ BA + 0.1 and 0.2 mg L^−1^ NAA + 1 g. L^−1^ combined activated charcoal, coconut milk, casein hydrolysate. The histological studies confirmed the somatic embryogenesis in purple coneflower. Generally, it was found that the somatic embryogenesis of *E. purpurea* occurs at high levels of BA and low levels of NAA with the addition of coconut milk and casein hydrolysate.

## Introduction

Purple coneflower (*Echinacea purpurea* (L.) Moench) is a perennial plant of the Asteraceae family that is widely used in the pharmaceutical, cosmetic and health industries. The medicinal products derived from the plant root and shoots are used to prevent and treat the colds, coughs, bronchitis, pulmonary infections, and chronic immunodeficiency diseases owing to the immune-enhancing properties [1].

The in vitro propagation of medicinal plants is significantly related to the production of high quality plant materials and pathogen-free plants and increasing number of plant species [2]. In addition, the in vitro techniques can facilitate the genetic manipulation of secondary metabolites production [3, 4]. The cultivation in an optimized medium under the controlled in vitro conditions is a viable alternative to the ex vitro cultivation of purple coneflower [5, 6]. The regeneration of *E. purpurea* can occur through the embryogenesis followed by the organogenesis [3, 5, 6]. Somatic embryogenesis is one of the advanced techniques for the mass plant propagation from somatic (asexual) cells in vitro [7]. Somatic embryogenesis is a suitable propagation method due to the further production and continuous proliferation of the embryogenic mass [8]. Also, in some cases, it is superior to other methods of asexual propagation, as it allows the mass propagation of plants using the bioreactors [9, 10]. Plant growth regulators (PGRs) play an important role in the induction and development of somatic embryogenesis [11]. Auxin is often required for the induction of somatic embryogenesis from different explants, and the process of somatic embryogenesis is generally initiated on the culture medium supplemented with high concentrations of auxins [11]. However, somatic embryogenesis has been induced on auxin-free media in various plants [12–14]. In several plants, a combination of auxin and cytokinin stimulates the development of somatic embryogenesis [11]. The use of 2,4-D (4.5 μM) and BA (0.45 μM) for the induction of somatic embryogenesis has been reported for yacon [*Smallanthus sonchifolius* (Poepp. and Endl.) H. Robinson] [15].The sensitivity of the explant tissue to the PGRs may have been altered by the dark treatment, thereby resulting in a higher frequency of embryogenesis [12, 16].

The somatic embryogenesis of *E. purpurea* was first observed by Choffe et al. [17] in the petiole explants cultured in the MS [18] medium containing 5 μM 6-Benzyl Amino Purine (BAP), Thidiazuron (TDZ), or TDZ and Indole-3-acetic acid (IAA). The histological observations of the cultures showed that the protoderm was well made of rectangular cells and there was no evidence of vascular association with native vessels. In addition, the cultivation of purple coneflower leaf explants for 14 days in darkness in the MS medium containing the combination of cytokinin BA (5 μM) with auxin Indole-3-Butyric Acid (IBA) (2.5 μM) and then in the light resulted in the occurrence of somatic embryogenesis [16]. Ahmad et al. [19] investigated the effect of different levels of growth regulators on the somatic embryogenesis of purple coneflower. The highest average number of embryos (24.03 explant^−1^) was obtained in the MS medium containing vitamin B5 [20] with 5 μM BAP and 2.5 μM IBA.

Lema-Rumińska et al. [6] used the MS medium containing BAP and α-Naphthalene Acetic Acid (NAA) for the somatic embryogenesis of *E. purpurea* that had been kept in darkness for 14 days. To stimulate the shoot proliferation of the embryos, the MS medium without auxin but containing cytokinin kinetin was used. In another study, Lakshmanan et al. [21] used the hypocotyl of four species of *Echinacea* (*E. purpurea, E. pallida, E. paradoxa* and *E. angustifolia*) for the induction of somatic embryogenesis in the MS medium containing 9 μM 3,6-dichloro-o-anisic acid (dicamba, DC) or 2*,*4*-*Dichlorophenoxyacetic acid (2,4-D). The embryos were obtained from all the cultures. *E. pallida* and *E. angustifolia* showed better embryogenesis than *E. paradoxa* and *E. purpurea*. The results were in contrast to the culture of the *E. purpurea* petiole [17] where 2,4-D inhibited the embryogenesis.

Since different concentrations of growth regulators have different effects on the somatic embryogenesis of *E. purpurea*, the present study was conducted to examine the effect of different growth regulators on the somatic embryogenesis and shoot induction in the leaf explants of *E. purpurea.* Moreover, to find out more about the microscopic changes during the embryogenesis, the embryogenic calli were histologically evaluated. Also, for the biochemical analysis, the protein concentration and the catalase and peroxidase activity of embryogenic, organogenic and undifferentiated calli were measured and finally, the survival rate of *E. purpurea* somatic embryos in the ex vitro conditions was evaluated.

## Materials and methods

### Establishment of aseptic seedlings

The seeds of *Echinacea purpurea* (L.) Moench were purchased from Pakan Bazr Company (Isfahan, Iran), washed using the sterile distilled water, and then were transferred to a solution containing 0.05% citric acid + 0.1% mercury chloride and disinfected for three minutes. Finally, they were transferred to the 0.05% citric acid solution and washed for three minutes. The seeds were placed in the half-strength (1/2) MS medium containing 7 g l^−1^ agar and 30 g l^−1^ sucrose at pH = 5.7. The culture medium was sterilized in an autoclave at 121 °C and 1.2 kg cm^−2^ for 20 min. The glass jars of 10 cm height and 6 cm diameter were used for the seed cultivation and eight seeds were placed in each jar. After the cultivation, the jars were incubated in the growth chamber with 16:8 h light /dark period, the temperature of 23 ± 2 °C in the light and 18 ± 2 °C in the dark period and the light intensity of photosynthetic photon flux density (PPFD) of 34–40 μmol m^−2^ s^−1^.

### Somatic embryogenesis and plant regeneration

**Fig. 1:**
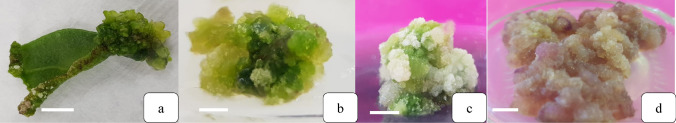
Different calli types developing on leaf explants of *E. purpurea* cultured on MS medium after four weeks: **a** Dark green and compact embryogenic calli induced by 3 mg L^−1^ BA and 0.5 mg L^−1^ NAA combination, 1 g L^−1^ active charcoal, 50 ml L^−1^ coconut milk and 50 mg L^−1^ casein hydrolysate after four weeks on MS medium. **b** Light green, soft, loose and puffy organogenic calli induced on MS medium supplemented with 3 mg L^−1^ BA and 0.5 mg L^−1^ NAA after four weeks. **c** White and light undifferentiated and hyperhydrated calli induced on MS medium supplemented with 3 mg L^−1^ BA and 0.1 mg L^−1^ NAA after four weeks. and **d** Browned and dark undifferentiated calli induced on MS medium supplemented with 3 mg L^−1^ BA and 0.2 mg L^−1^ NAA after four weeks. *Bars* = 10 mm

Six weeks after the seed culture, the 1 × 1 cm leaf explants were isolated from the plantlets of *E. purpurea* and cultivated in the MS medium containing 30 g. L^−1^ sucrose and 7 g. L^−1^ agar with the pH of 5.7 containing different growth regulator compounds provided by BA and NAA Table 1. In this experiment, the interaction of different levels of BA (3, 4 and 5 mg L^−1^) and NAA (0.1, 0.2 and 0.5 mg L^−1^) with or without 1 g L^−1^ active charcoal, 50 ml L^−1^ coconut milk and 50 mg L^−1^ casein hydrolysate were evaluated Table 1. To induce embryogenic callus formation from the leaf explants, the jars were incubated in the dark for 14 days and then transferred to the growth chamber in the aforementioned conditions. After four weeks, the explants began to develop embryos or organogenesis in the callus of leaf explants, and the percentage of organogenic and embryogenic calli, as well as those that failed to regenerate were measured. After eight weeks, the embryos began the organogenesis (root and shoot formation) (Fig. 1). At this time, the number of leaves was counted. The regenerated plants were removed with the attached callus and transferred to the MS medium containing 0.1 mg L^−1^ BA for the shoot proliferation (Fig. 1).

**Table 1:** Different treatments for somatic embryogenesis induction in *E. purpurea*

Treatments	T1	T2	T3	T4	T5	T6	T7	T8	T9	T10	T11	T12	T13	T14	T15	T16	T17		T18
BA ( mg L^−1^)	3	3	3	4	4	4	5	5	5	3	3	3	4	4	4	5	5		5
NAA ( mg L^−1^)	0.1	0.2	0.5	0.1	0.2	0.5	0.1	0.2	0.5	0.1	0.2	0.5	0.1	0.2	0.5	0.1	0.2		0.5
Casein hydrolysate ( mg L^−1^)	0	0	0	0	0	0	0	0	0	50	50	50	50	50	50	50	50		50
Active charchoal (mg L^−1^)	0	0	0	0	0	0	0	0	0	1	1	1	1	1	1	1	1		1
Coconut milk ( ml L^−1^)	0	0	0	0	0	0	0	0	0	50	50	50	50	50	50	50	50		50

### Adaptation and acclimatization of plantlets

**Fig. 2:**
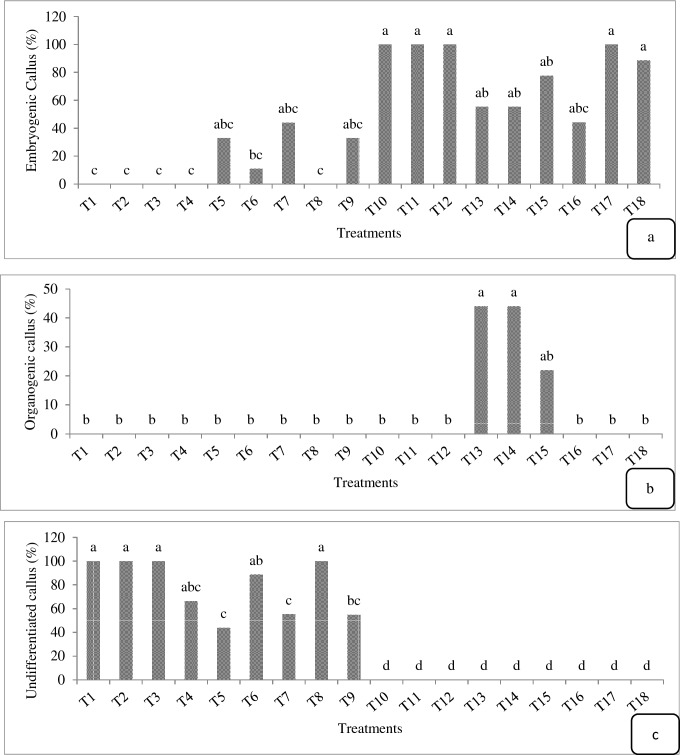
Effect of different treatments on induction of different calli types developing on leaf explant calli of *E. purpurea* cultured on MS medium after four weeks **a** embryogenic, **b** organogenic, and **c** undifferentiated calli

The root initiation occurred in the base of shoot explants in the MS medium containing 0.1 mg L^−1^ BA after six weeks. After six weeks, the rooting, leaf chlorophyll, root length, number and length of plantlet leaves were measured. The plantlets were transferred for the acclimatization after the initial preparation in the culture medium to the pots containing 70% peat moss and 30% perlite, which were previously moistened with the distilled water and soaked with the benomyl fungicide at a ratio of 1.5:1000. The culture substrate was poured into the plastic pots with a diameter of 7 cm. A sub-irrigation system was used to irrigate the pots (Fig. 2). A transparent plastic cup was placed on the top of each pot and was removed after two weeks (Fig. 2). Six weeks after transferring the plants to the pots, the percentage of acclimatized plants was measured.

### Measurement of protein, catalase and peroxidase

The embryogenic callus culture in the fourth week and the organogenic and undifferentiated callus culture in the eighth week were used to measure the protein concentration and the catalase and peroxidase activity. To measure the protein, the Bradford reagent was first prepared and then, the protein was extracted [22].

To measure the activity of peroxidase, 2 ml of reaction mixture including 50 mg of protein (this value calculated using standard curve), 5 mM guaiacol and a sufficient amount of 25 mM phosphate buffer (pH = 7) were mixed to reach the final volume of 2 ml. The spectrophotometer (CECIL 9500, England) was zeroed at the wavelength of 470 nm using this mixture, and then 5 µl of 30% hydrogen peroxide (H_2_O_2_) was added to this mixture, and the light absorption changes were rapidly measured for one minute at a time interval of 10 s. The amount of enzyme activity was expressed in terms of light absorption changes per minute per milligram of protein (ΔOD/min/mg protein) [23].

To measure the activity of catalase, the reaction mixture included 50 mM potassium phosphate buffer (pH = 7) and 15 mM hydrogen peroxide. The reaction was started by adding 100 µl of the enzyme extract to a final volume of 3 ml. The absorption changes were recorded at 240 nm for three minutes based on the millimolar of hydrogen peroxide per milligram of protein (ΔOD/min/mg protein) [24].

### Histological study

**Fig. 3:**
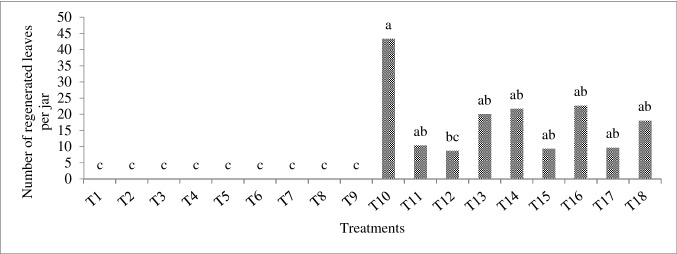
Effect of different treatments affecting number of regenerated leaves of *E. purpurea* cultured on MS medium after eight weeks

The calli and morphology of the embryos were visually inspected (Fig. 3). The histological studies were performed to evaluate the somatic embryogenesis, where the embryogenic calli were placed for 24 h in the fixative FAA (formaldehyde, acetic acid and 100% ethanol solution with ratios of 17:1:2). After washing, they were dehydrated with the increasing degrees of ethanol and finally saturated with toluene-paraffin mixture and then pure paraffin. The samples were molded in paraffin and the 7–8 μm tissue slices were prepared using a microtome. The slices were glued onto the glass slides and the toluene was used as a paraffin solvent. After removing the paraffin, hematoxylin–eosin was used for staining the samples. Then, the slides were observed by light microscope (Eclipse 80i, Nikon, Japan) and the samples were photographed.

### Experimental design and statistical analysis

The experiment was conducted in a completely randomized design with three repetitions. Three leaf explants were cultured on each jar and each jar was considered as a single repetition. The analysis of variance was performed for all data using SAS 9.1 statistical software and the Duncan's multiple range test (DMRT) was used for the comparison of means at 5% probability level.

## Results

**Fig. 4:**
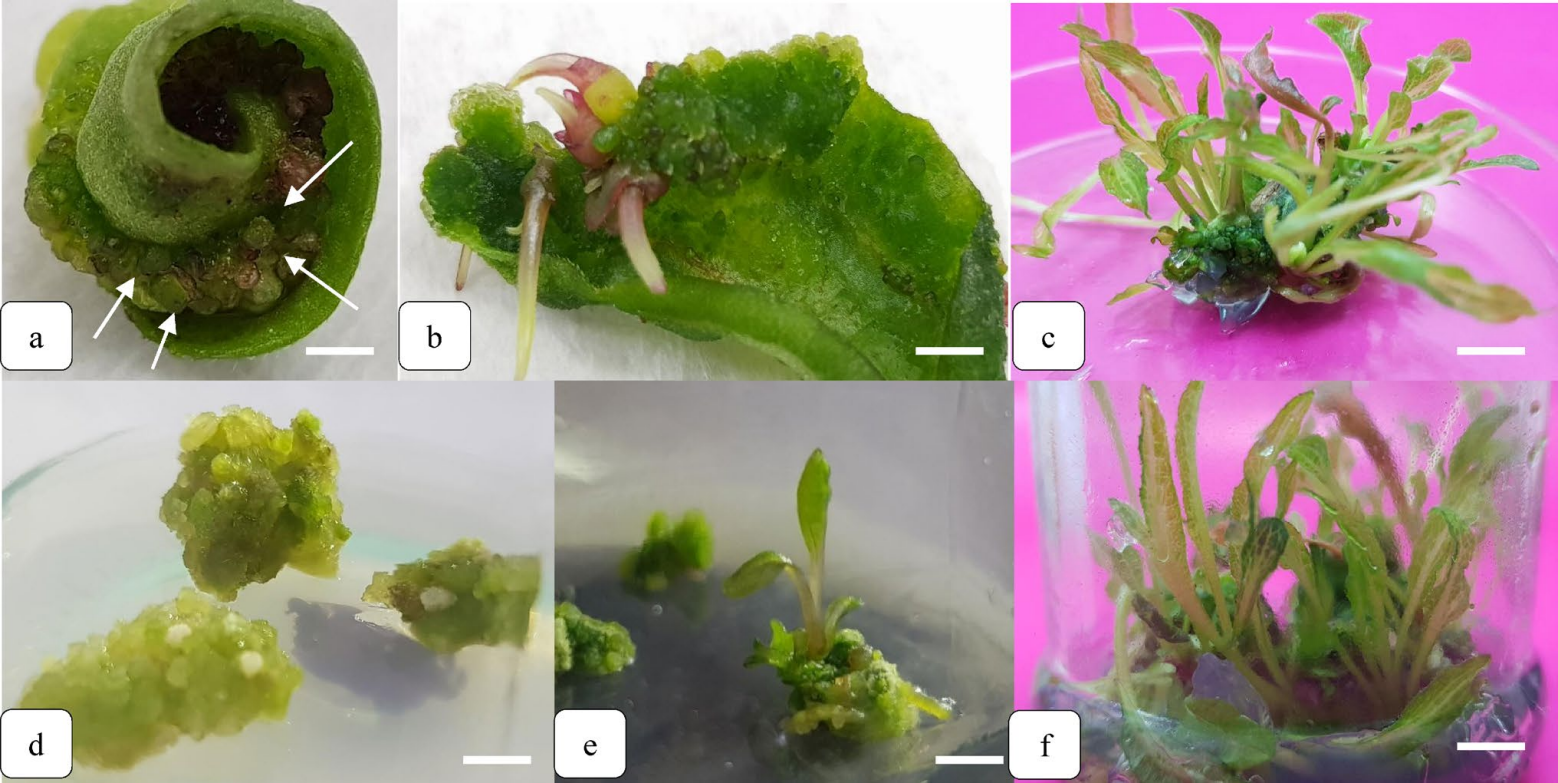
Production steps of plantlet in *E. purpurea* **a** somatic embryos (*white arrows*) regenerated from leaf explant callus of *E. purpurea* derived from combination of 3 mg L^−1^ BA and 0.5 mg L^−1^ NAA with 1 g L^−1^ active charcoal, 50 ml L^−1^ coconut milk and 50 mg L^−1^ casein hydrolysate cultured on MS medium. *Bars* = 10 mm, **b** shoots regenerated from somatic embryos. *Bars* = 10 mm, **c** leaf production from embryogenic callus. *Bars* = 20 mm, **d** organogenic calli. *Bars* = 10 mm, **e** shoot regenerated from organogenic callus. *Bars* = 10 mm, and **f** microshoot production from organogenic callus. *Bars* = 20 cm

In the present study, the calli were classified into three groups: undifferentiated, embryogenic and organogenic (Fig. 3). The embryogenic calli were dark green and coherent with a faster growth rate (Fig. 3a). The use of activated charcoal, casein hydrolysate, and coconut milk combined with growth regulator treatments significantly increased callus embryogenesis compared to the treatments only with BA and NAA combination (Fig. 4a). The highest share of explants with embryogenic calli (100%) were observed in T10, T11, T12, T17 and T18 treatments (88.7% of explants) (Fig. 4a). The somatic embryos were also observed in T5 and T9 (33% of explants), T6 (11% of explants), and T7 (44% of explants) treatments (Fig. 4a). The combination of BA and NAA treatments without coconut milk and casein hydrolysate failed to produce embryos in T1, T2, T3, T4 and T8 treatments (Fig. 4a). The organogenic calli were observed in T13, T14 and T15 treatments (20–44% explants) (Fig. 4b). The calli were soft, loose and puffy in light green with low growth rate (Fig. 3b).

The combined treatments of NAA and BA without any additives (activated charcoal, casein hydrolysate and coconut milk) produced undifferentiated soft callus (in 50% – 100% of explants) (Fig. 4c). As such, the addition of casein hydrolysate, coconut milk and activated charcoal caused both differentiation and embryogenesis of calli, and in the free media of these compounds (casein hydrolysate, coconut milk and activated charcoal), the undifferentiated callus was observed. The highest amount of undifferentiated calli (100%) was observed in T1, T2, T3 and T8 treatments (Fig. 4c). The undifferentiated calli were white and light (Fig. 3c) or brown and dark (Fig. 3d) and appeared after six weeks.

**Fig. 5:**
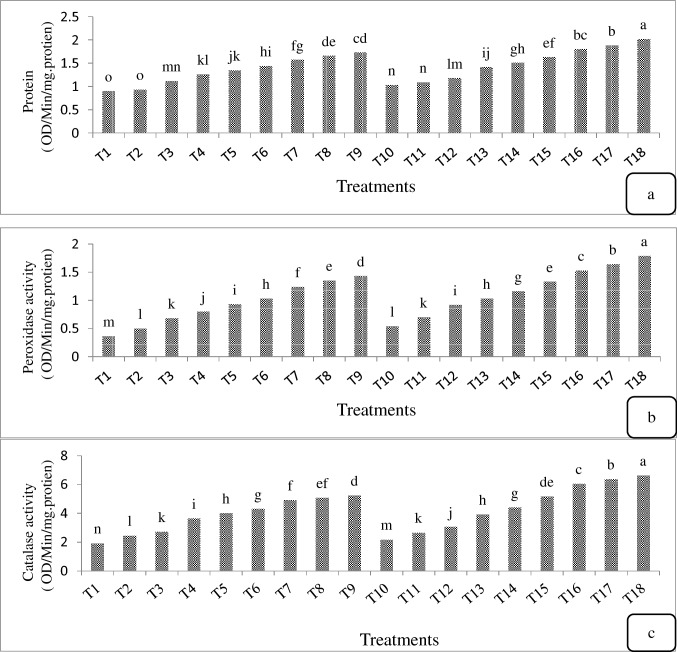
Effect of different treatments affecting **a** protein content, **b** activity of catalase, and **c** activity of peroxidase in calli of *E. purpurea*

After eight weeks of in vitro culture, the calli began to regenerate leaves (Figs. 5, 1). The results showed that in all of the treatments containing a combination of BA and NAA growth regulators, the embryos failed to regenerate and produce leaves (Fig. 5). However, in all treatments containing the activated charcoal, coconut milk and casein hydrolysate (together with BA and NAA), the embryos were regenerated and produced the leaves (Figs. 5 and 1). The highest leaf number (43.3 per jar) was observed in T10 treatment (Fig. 5). The leaves were produced on the both embryogenic and organogenic calli (Fig. 1b–f).

**Fig. 6:**
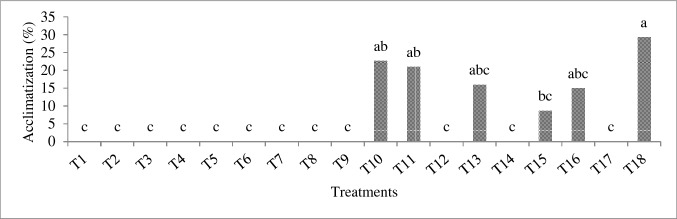
Effect of different treatments on acclimatization of *E. purpurea* plantlets six weeks after transferring plants to pots

The protein content and the activity of catalase and peroxidase in callus were evaluated in all treatments (Fig. 6). The highest amount of protein and the peroxidase and catalase activity (2.02, 1.79 and 6.62 ΔOD/min/mg. protein) were obtained in T18 treatment (Fig. 6). Based on the results, with increasing the BA and NAA concentration, the protein content and the peroxidase and catalase activity of calli significantly increased (Fig. 6). The lowest amount of protein was obtained in T1 and T2 treatments (0.9 and 0.93 ΔOD/min/mg. protein) (Fig. 6a) and the lowest peroxidase and catalase activity in T1 (0.36 and 1.92 ΔOD/min/mg. protein) (Fig. 6b and c).

The plantlet production and rooting occurred only in T10, T11, T13, T15, T16 and T18 treatments (Table 2). The failure to use casein hydrolysate, activated charcoal and coconut milk did not lead to the plantlet regeneration and production (Table 2). The highest root length was obtained in T10 (63.5 mm), T11 (78.7 mm), T13 (60.1 mm) and T18 (53 mm) treatments (Table 2). The maximum shoot height after removing from the in vitro culture medium and before the acclimatization was obtained in T10 (78.7 mm), T11 (102 mm), T13 (92.2 mm) and T18 (73.9 mm) treatments (Table 2). The highest leaf length (38.9 mm) was obtained in T15 treatment (Table 2). The highest amount of chlorophyll was obtained in T10 (33.3 SPAD value), T11, T13 (28.4 SPAD value) and T18 (26.6 SPAD value) treatments (Table 2).

**Table 2:** Effect of different treatments on some growth traits of purple coneflower plantlets after removing from in vitro culture medium and before acclimatization

Characteristics	T1	T2	T3	T4	T5	T6	T7	T8	T9	T10	T11	T12	T13	T14	T15	T16	T17	T18
Length of Roots (mm)	0^b^	0^b^	0^b^	0^b^	0^b^	0^b^	0^b^	0^b^	0^b^	63.45^a^	78.84^a^	0^b^	60.13^a^	0^b^	24.92^b^	19.99^b^	0^b^	52.95^a^
Height of plantlets (mm)	0^b^	0^b^	0^b^	0^b^	0^b^	0^b^	0^b^	0^b^	0^b^	87.71^a^	102.49^a^	0^b^	92.19^a^	0^b^	19.01^b^	33.91^b^	0^b^	73.85^a^
Length of biggest leaf (mm)	0^b^	0^b^	0^b^	0^b^	0^b^	0^b^	0^b^	0^b^	0^b^	27.04^ab^	27.01^ab^	0^b^	31.84^ab^	0^b^	38.94^a^	11.87^ab^	0^b^	27.07^ab^
Cholorophyll (SPAD value)	0^b^	0^b^	0^b^	0^b^	0^b^	0^b^	0^b^	0^b^	0^b^	33.33^a^	28.36^a^	0^b^	28.40^a^	0^b^	13.06^b^	4.30^ab^	0^b^	26.56^a^

**Fig. 7:**
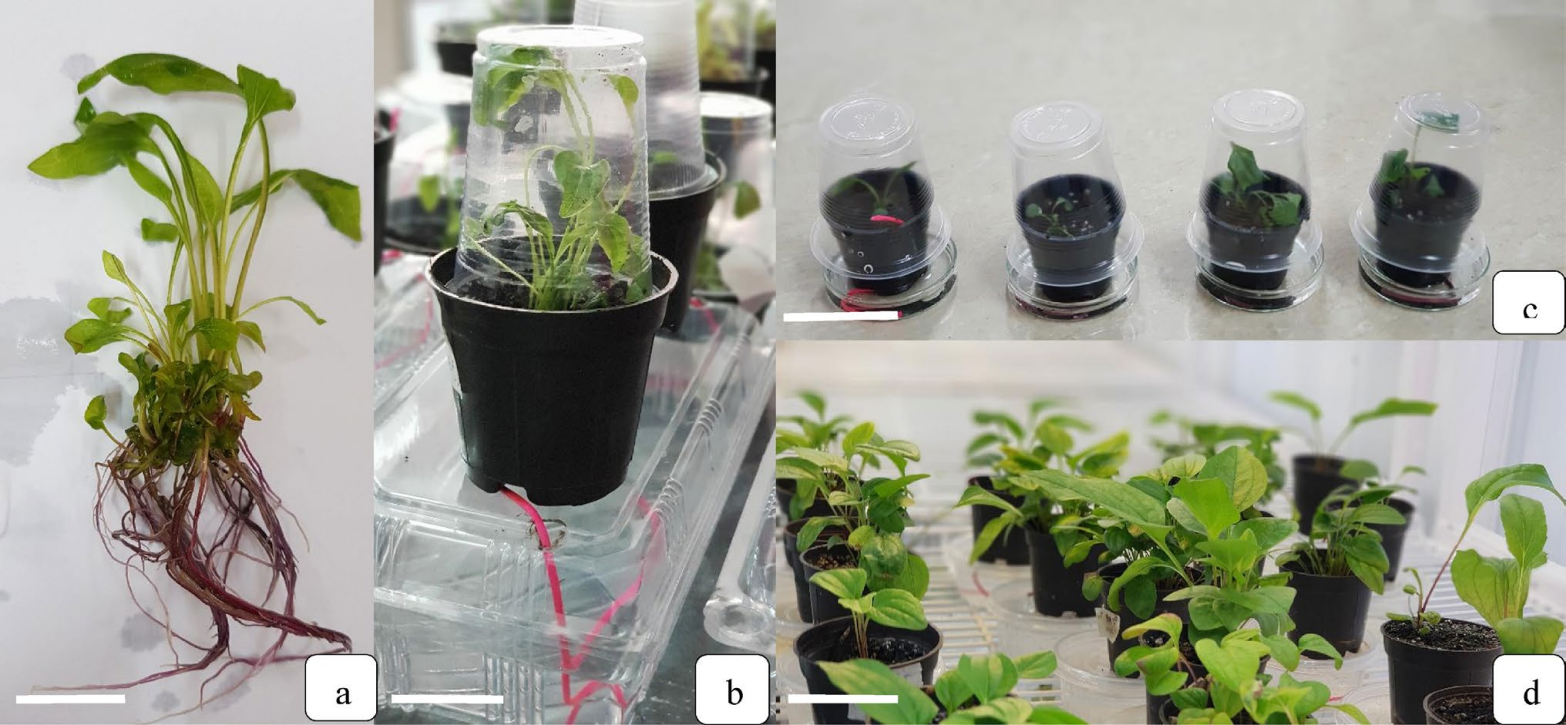
Different adaptation stages of plantlets **a** plantlets obtained from embryogenic callus. **b** plantlets transferred to small pots containing 70% peat moss and 30% perlite for acclimatization and irrigation of pots by underground (sub) irrigation method. **c** sample plantlets during transfer to pots. and **d** plantlets after four weeks of acclimatization. *Bars* = 1 cm

The percentage of acclimatized plantlets six weeks after transferring the plants to the pots is shown in Fig. 2. Only the plants obtained from T10 (22.7% of plants), T11 (21%), T13 (16%), T15 (8.66%), T16 (15%) and T18 (29.3%) treatments survived the acclimatization (Fig. 7). The highest percentage of acclimatization success (29.3% of plants) was observed in T18 treatment (Fig. 7).

**Fig. 8:**
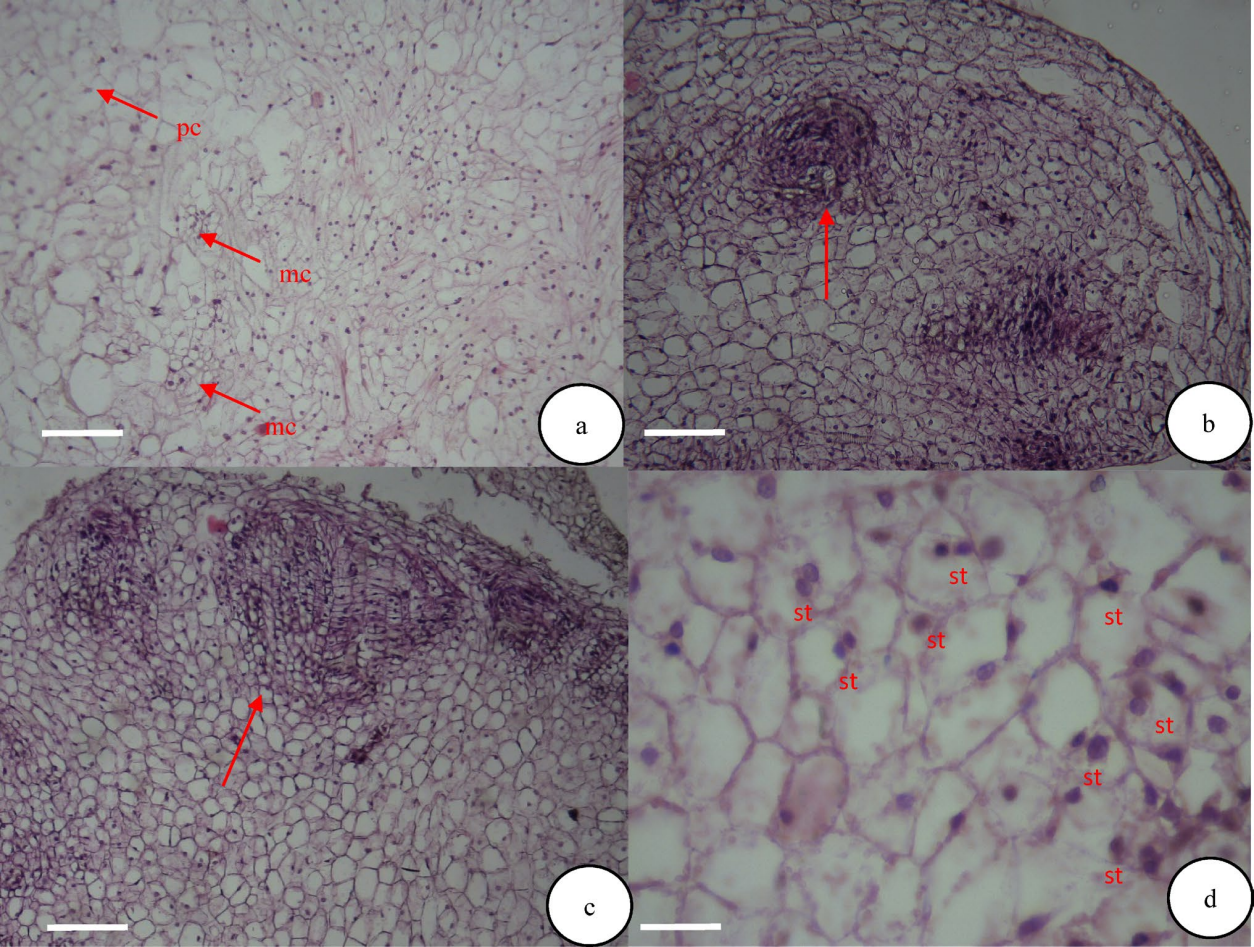
Histological study of embryogenic and organogenic calli in *E. Purpurea* after four weeks; **a** parenchymal (pc) and meristematic (mc) cells in organogenic callus induced on MS medium supplemented with 3 mg L^−1^ BA and 0.5 mg L^−1^ NAA after four weeks, *Bars* = 1 µm, **b** embryogenic callus and presence of globular embryo (*red arrows*) induced by 3 mg L^−1^ BA and 0.5 mg L^−1^ NAA combination, 1 g. L^−1^ active charcoal, 50 ml L^−1^ coconut milk and 50 mg L^−1^ casein hydrolysate after four weeks on MS medium, *Bars* = 5 µm, **c** embryo in heart stage (*red arrows*) after five weeks on MS medium, *Bars* = 5 µm, **d** embryonic cells of dense cytoplasm, large nucleus and starch (st) granules, *Bars* = 10 µm

The histological studies confirmed the different stages of somatic embryogenesis. The different embryogenic stages were visible in the embryogenic masses, which were the pro-embryo, globular, heart, torpedo and cotyledon stages, and the heart and globular stages were histologically studied (Fig. 8 b and c). The non-embryonic cells were large with small nuclei and low cytoplasm density compared to the embryonic cells (Fig. 8a). In contrast, the embryogenic cells were compact and small with large nuclei and dense cytoplasm (Fig. 8b and c).

The histological study revealed that the organogenic calli were characterized by prominent parenchymal cells without a distinct nucleus (Fig. 8a). The meristem cells were smaller than the surrounding cells (Fig. 8a). Figure 8b shows the globular embryonic masses within the callus cells, and the heart embryos were formed by continuing the cell divisions (Fig. 8c). The differentiated leaves were formed from the surface of organogenic calli, showing vascular association with leaf explant. The cells in the embryogenic calli showed dense cytoplasm, large nuclei, and starch storage granules (Fig. 8d). The cell proliferation was also observed in the embryogenic callus tissue.

On the margins of the embryogenic callus, the areas with meristematic activity were visible, where the successive divisions resulted in the development of somatic embryos (Fig. 8c). The embryonic cells in the slices were small, compacted cells whose cytoplasm was highly stained and clearly had high nucleus volume. The embryos were not easily detachable from the callus tissue. With the cell divisions at the two embryo poles, the globular embryos gradually began to stretch toward the bipolarity, and thus, the basic structure of cotyledons was developed and the embryos took a heart form (Fig. 8c).

The successive divisions at the two embryo poles led to the development of embryos and eventually, to the emergence of mature cotyledons where the cotyledons were fully grown. Gradually, the differentiation of cells in the formation of vascular bundles was observed in the cotyledon embryos, and the mature embryos were separated from the native cells by appropriate growth, placed on the germination medium, and produced leaves and roots (Fig. 1 b and c).

## Discussion

The results of the present study showed that the organogenesis and somatic embryogenesis of the purple coneflower as a medicinal-ornamental plant can be easily regulated by the use of BA, NAA, coconut milk, activated charcoal, and casein hydrolysate. In the indirect organogenesis, the combination of BA and NAA was effective in the shoot induction. The highest production of organogenic callus (44% of explants) was obtained in 4 mg L^−1^ BA and 0.1 or 0.2 mg L^−1^ NAA + coconut milk + activated charcoal + casein hydrolysate supplemented medium. The organogenesis of calli began three weeks after the establishment of leaf explant culture. The results were consistent with those of Zhang et al. [25] on *Lilium pumilum* DC. They reported that the shoot induction rate increased with increasing the BA/NAA ratio and 92.5% of the scales were managed to produce the shoots after 6 weeks in the MS medium containing 2 mg L^−1^ BA and 0.2 mg L^−1^ NAA. One of the reasons may be due to the more effective role of BA than NAA in differentiating the cambium [26]. Bakhshaie et al. [27] obtained the highest percentage (65.5%) of somatic embryogenesis in *Lilium ledebourii* in the medium containing 0.54 μM NAA and 0.44 μm BA. Auxin seems to cause further DNA methylation than normal and may be required for the reprogramming of differentiated cells [28]. Hence, the tissue-specific programs, especially with differentiation, can be eliminated by hypermethylation, reaching the final site of differentiation perhaps with a small fragment of cells, and eventually will be capable of organogenesis or embryogenesis [28].

It is important to note in this study that the somatic embryos were indirectly produced. The callus was first developed on some leaf explants of purple coneflower and then, the shoots emerged from the embryogenic calli. As a result, the vascular relationship between shoots and plant tissue (explant) was fairly clear in the embryogenic callus mass. In other parts, the lower epidermis cells were undifferentiated and the somatic embryos were produced in the epidermal cells. The obtained results were consistent with those of Choffe et al. [17]. In a histological analysis Correa et al. [15], revealed that embryogenic calli were more friable and yellowish in appearance than the non-embryogenic calli. However, the embryogenic calli *of E. purpurea* were dark green and coherent with a faster growth rate than the non-embryogenic calli. The organogenic calli were light green, soft, loose and puffy with low growth rate.

In this study, the somatic embryos were obtained from the leaf explant in purple coneflower. The positive effect of adding casein hydrolysate to culture medium was determined for the differentiation of somatic embryo in purple coneflower. Therefore, by adding casein hydrolysate to culture medium, the somatic embryo production was promoted. Our observations are consistent with the findings of Mauro et al. [29] and Mahmood et al. [30] on the grape. Although the treatments (T5, T6, T7, and T9) lacking casein hydrolysate, coconut milk and activated charcoal produced embryogenic calli, they were not able to regenerate the leaves. In addition, the T12, T14 and T17 treatments with the above-mentioned compounds failed to regenerate the leaves. The regenerated plants began to acclimatize after moving to the ex vitro medium. The T10 (22.7%), T11 (21%) and T18 (29.3%) treatments showed the highest acclimatization efficiency in this respect. The presence of casein hydrolysate and activated charcoal had a positive effect on the embryogenesis, as the results showed that beside the use of plant growth regulators, the treatments containing casein hydrolysate, activated charcoal and coconut milk showed significantly higher embryogenesis than the media without these compounds. Casein hydrolysate contains the amino acids necessary for the embryogenesis, such as glutamine, proline, alanine, serine and glycine. The proline and serine amino acids increase the mitotic activities of cells by increasing the levels of internal hormones, thereby enhancing the development of early embryonic cell masses [31]. The organic amino acids in casein hydrolysate can be a good substitute for the inorganic ammonium and a supplement to nitrate. Also, the nitrogen uptake through an organic source such as casein hydrolysates is much easier and faster than that through an inorganic source [32]. The activated charcoal is composed of carbon arranged in a quasi-graphitic form in small particle size. It is often used in the medium to improve the growth and development of cell, tissue, and organ [33]. The activated charcoal has the potential to absorb some inorganic ions, auxins, cytokinins, and phenolics. The positive effect of activated charcoal on embryo maturation and conversion was probably caused by the adsorption of PGRs [34].

The endosperm products, especially coconut milk, have cytokinin activities. These natural products have a reduced nitrogen source and a range of complex chemical compounds, which are capable of stimulating the growth and organogenesis. The analysis of coconut milk has shown that there are different oligosaccharides some of which have growth regulating activities [35].

The somatic embryogenesis and the direct or indirect organogenesis are mainly used as the micro-propagation techniques in the plant tissue culture [36]. In addition, the embryogenic callus is a suitable receptor tissue for the genetic changes [37]. Somatic embryogenesis is still commonly believed to be the most efficient micro-propagation technique in genetic transformation [38]. The organogenic calli were light green, soft and translucent and produced the leaves in response to NAA and BA. They mainly contained the parenchymal cells and produced the shoot, which showed a vascular relationship with the leaf explant. However, the embryogenic calli were dark green, had a strong spherical structure, and developed in response to the combination of BA and NAA as well as coconut milk, activated charcoal and casein hydrolysate. The embryos had dense cytoplasm, large nuclei, and storage proteins. The results were consistent with the histomorphological studies on *Lilium pumilum* [26], *Pulsatilla koreana* [39] and *Zea mays* [40]. According to the totipotency theory, every living plant cell has the potential to regenerate into a complete plant with the somatic embryogenesis [41]. However, not all embryogenic callus cells are capable of producing somatic embryos in *E. purpurea*. Thus, the shoot and root meristematic centers of somatic embryos play an important role in improving the efficiency of somatic embryogenesis [42]. Soundar Raju et al. [43] stated that the appearance of protoderm is an indicator of true somatic embryogenesis. Also, at the induction stage (globular somatic embryo stage) of garlic (*Allium sativum* L.) [44] and turmeric (*Curcuma longa* L.) [43], the appearance of protoderm was an indicator of somatic embryogenesis.

The in vitro production of callus tissue is usually accompanied by the problem of callus browning due to the accumulation of phenolic compounds and subsequent oxidation (enzymatic or non-enzymatic). Callus browning (callus tissue necrosis) has caused a number of serious problems, and thus, it is critical to produce healthy, rapidly growing callus in the in vitro studies to extract secondary metabolites and subsequently, apply on a large scale [45]. The callus browning in tissue culture is affected by different factors such as species, explant, culture conditions, physiological state, pretreatments, medium composition, temperature, and subculture frequency [46, 47]. It has been shown that transferring to fresh medium, activated charcoal, polyvinylpyrrolidone (PVP), silver nitrate, and ascorbic acid is used to overcome the in vitro browning and to remove phenols or reduce their accumulation in the culture media [47].

The callus protein content varied according to the concentration of growth regulators and the presence of casein hydrolysate and coconut milk. For example, the highest amount of protein was obtained at the highest concentration of BA and NAA with casein hydrolysate, coconut milk and activated charcoal, while the lowest amount was obtained in the medium containing the lowest level of BA and NAA and lacking these organic additives. Enzymes are made of protein and the total protein content can be profiled in the biologically active site of a cell. Plants have numerous defense systems, including the increased activity of antioxidant enzymes to remove free radicals and reduce oxidative stress [48]. Antioxidant enzymes play an important role in scavenging free radicals (O_2_^–^, H_2_O_2_ and OH^–^). They are naturally produced during the metabolic activity, but under the unfavorable conditions, a burst occurs in their production that results in the poor performance such as protein catabolism (proteolysis), DNA mutation, membrane peroxidation, and eventually plant death. In plants, both enzymatic and non-enzymatic processes are involved in the detoxification of reactive oxygen species (ROS) which can lead to the oxidative damage to many cell compartments such as membrane lipids, proteins, and nucleic acids [49–51]. The free radical scavenging enzymes such as superoxide dismutase, ascorbic acid peroxidase, catalase, and guaiacol peroxidase scavenge the free radicals and reduce the cell degradation [52]. The evaluation of catalase and peroxidase activity showed that the elevated levels of BA significantly increased the activity of both antioxidant enzymes. In fact, the results of this study showed that the increase in the levels of growth regulators improved the protein content and the antioxidant enzyme activity. Moreno et al. [53] studied the peroxidase activity in the callus culture of radish (*Raphnus sativus* cv. Cherry Bell). The callus induction occurred in different combinations of BA and 2,4-D in the MS medium. They found that the activity of peroxidase on the callus of all explants was higher than that of the intact plant. Shank et al. [54] examined the peroxidase activity in the calli of different explants of *Moringa oleifera*. They also showed that the enzyme activity was higher in callus than in plant.

In the micro-propagation system of plant, the ex vitro acclimatization, or hardening, is one of the main processes performed for the production of healthy plantlets before their transplantation to field conditions [55]. The in vitro-developed plantlets of *E. purpurea* were acclimatized after transplanting with an 8.66–29.3% survival rate. Sivanesan and Jeong [11] reported that 98% of cineraria plantlets were successfully acclimatized in the greenhouse. The in vitro conditions including low light intensity, levels of sucrose and other nutrients, and high relative humidity may cause physiological and anatomical changes that have negative effects on the acclimatization of regenerated plants and explain the limited acclimatization efficiency in this study [56].

During the ex vitro acclimatization, many changes may occur in the physiological and morphological metabolisms such as photosynthesis due to the differences in the environmental conditions [55]. The highest amount of chlorophyll content was obtained in the MS medium supplemented with BA, NAA, 1 g L^−1^ active charcoal, 50 ml L^−1^ coconut milk and 50 mg L^−1^ casein hydrolysate in T10, T11, T13 and T18 treatments. It has been reported that the increased growth of plantlets is a consequence of the increased photosynthetic rate of the plantlet due to the control of environmental conditions during the in vitro culture, which also affects the plantlet growth and survival during the ex vitro acclimatization [57, 58]. Among different treatments, it seems that T10 and T11 treatments showed the best growth characteristics during the ex vitro acclimatization.

## Conclusion

In this study, a simple and reproducible process was developed for the somatic embryogenesis and plant regeneration of *E. purpurea.* The results of this study showed that a combination of cytokinin BA (at high concentrations) and auxin NAA (at low concentrations) in the presence of coconut milk, activated charcoal and casein hydrolysates had a positive effect on the somatic embryogenesis in the leaf explants of *E. purpurea.* The histological studies confirmed the different stages of somatic embryogenesis. The biochemical analysis results revealed that with increasing the BA and NAA concentrations, the protein content and the peroxidase and catalase activity of calli significantly increased. The in vitro-developed plantlets of *E. purpurea* were successfully acclimatized after transplanting with an 8.66–29.3% survival rate. The highest amount of chlorophyll content in the acclimatized plantlets was obtained in the MS medium supplemented with BA, NAA, 1 g. L^−1^ active charcoal, 50 ml L^−1^ coconut milk and 50 mg L^−1^ casein hydrolysate. These results serve as the initial step to optimize culture media for a variety of purposes, such as in vitro propagation, conservation, metabolite extraction, plant breeding, genetic transformation or applied biotechnology, including the use of synthetic seeds or bioreactors.
